# Ti-doped ZnO Thin Films Prepared at Different Ambient Conditions: Electronic Structures and Magnetic Properties

**DOI:** 10.3390/ma3063642

**Published:** 2010-06-09

**Authors:** Zhihua Yong, Tao Liu, Tomoya Uruga, Hajime Tanida, Dongchen Qi, Andrivo Rusydi, Andrew T. S. Wee

**Affiliations:** 1 Physics Department, Faculty of Science, National University of Singapore, 117542, Singapore; 2 Institute for Synchrotron Radiation, Karlsruhe Institute of Technology, 76344, Germany; 3 SPring-8/JASRI, Hyogo, 679-5198, Japan; 4 NanoCore, National University of Singapore, 117576, Singapore; 5 Singapore Synchrotron Light Source, National University of Singapore, 117603, Singapore

**Keywords:** DMS, XAFS, ZnO, vacancy, Ti

## Abstract

We present a comprehensive study on Ti-doped ZnO thin films using X-ray Absorption Fine Structure (XAFS) spectroscopy. Ti K edge XAFS spectra were measured to study the electronic and chemical properties of Ti ions in the thin films grown under different ambient atmospheres. A strong dependence of Ti speciation, composition, and local structures upon the ambient conditions was observed. The XAFS results suggest a major tetrahedral coordination and a 4+ valence state. The sample grown in a mixture of 80% Ar and 20% O_2_ shows a portion of precipitates with higher coordination. A large distortion was observed by the Ti substitution in the ZnO lattice. Interestingly, the film prepared in 80% Ar, 20% O_2_ shows the largest saturation magnetic moment of 0.827 ± 0.013 *µ_B_*/Ti.

## 1. Introduction

Spintronics, the manipulation of spin in electrons in semiconductors presents a new paradigm for versatile functionalities in electronic materials. Ferromagnetic semiconductors with Curie temperatures (*T_C_*) above room temperature are ideal for realizing efficient spintronics devices. Dilute magnetic semiconductors (DMS) are promising materials since they have both charge and spin degrees of freedom in a single matrix, leading to interplay of magnetic, optical, and electronic functionalities. However, the origin of room temperature ferromagnetism in DMS is still controversial. Many mechanisms have been proposed so far. For example, the carrier-induced ferromagnetism which includes the RKKY (Ruderman-Kittel-Kasuya-Yosida) model [1,2], the double exchange mechanism [3], and more recently, Coey *et al.* [4,5] and Chambers *et al.* [6] reported that the phantom ferromagnetism can result from structural defects. Coey *et al.* [7] and Venkatesan *et al.* [8] proposed the bound magnetic polaron model [9], which states that ferromagnetic exchange is mediated by shallow donor electrons, which form bound magnetic polarons. Interestingly, as proposed by Elfimov *et al.* [10] and Jorge *et al.* [11], cationic vacancies can also lead to ferromagnetism. The experimentally observed ferromagnetism is often plagued by possible magnetic precipitates or clusters in the host semiconductors, casting doubts upon the reproducibility and reliability of current DMSs. Hence, careful studies on the electronic and atomic structures of DMS are essential to identifying the origin of the ferromagnetism in DMS for future fabrication of high T_C_ ferromagnetic DMSs.

Semiconducting zinc oxide (ZnO) offers significant potential in providing charge, photonic, and spins based functionalities [12]. ZnO is also well known for its direct band-gap and large excitation energy, and exhibits unique piezoelectric and electro-optic properties with potential applications in UV photonics and transparent electronics. Since ZnO has been identified as a good host material for realizing wide band-gap DMS with high *T_C_* ferromagnetism by doping with transition metal ions, ZnO has been selected as the host material in our investigation. In particular, Ti-doped ZnO seems to be a promising DMS material [8,13,14], since neither Ti nor its oxides are ferromagnetic. However, previous reports on the magnetic properties of ZnO:Ti are inconsistent; the theoretical value of the magnetic moment of Ti doped in ZnO is predicted to be 0 by Sato *et al.* [15] However, Antony *et al.* [13] and Venkatesan *et al.* [8] reported a saturation moment of about 0.15 *µ_B_*/Ti atom at room temperature for 5% Ti-doped ZnO sample, and Osuch *et al.* [14] performed density functional theory calculations predicting a magnetic moment of 0.63 *µ_B_* per supercell in Zn_0.9375_Ti_0.0625_O.

X-ray absorption fine structure (XAFS) measurements reveal the absorption characteristics of X-rays absorbed by an atom at energies near and above the core-level binding energies of that atom. XAFS spectra give information on the immediate environment around each absorbing species, and are especially sensitive to the formal oxidation state, coordination chemistry, interatomic distances, coordination number, and the species of atoms immediately surrounding the selected element. As XAFS provides a practical and relatively simple way to determine the chemical state and local atomic structure for selected atomic species, we make full use of this technique to investigate the nature of the Ti ions doped in the ZnO matrix.

In this work, Ti-doped ZnO thin films were prepared at several ambient conditions and the structure, electronic state, composition, and chemical environment were investigated. The composition of the prepared films depends on the ambient atmospheres because of the different deposition rates. From the results, a possible link between the structural and electronic properties is explored and the mechanism leading to ferromagnetism in Ti-doped ZnO films is discussed.

## 2. Experimental Section

Ti-doped ZnO thin films were fabricated by a reactive radio-frequency (RF) magnetron sputtering from a commercial ZnO target of 99.995% purity with a small plate of pure Ti metal plate attached. A ZnO target is selected instead of a Zn metallic target because the control of film stoichiometry is easier with oxide targets, thus alleviating the need for high temperature and post-deposition annealing [16].

A ZnO buffer layer was first grown between the Si(100) substrate and ZnO film to reduce the lattice mismatch between ZnO and Si(100). Next, the ZnO layer was deposited at a higher temperature on the buffer layer to obtain a high-quality thin film. Reports [17,18,19] have shown that there is marked improvement in both the optical properties and crystalline quality achieved through a two-step growth using the RF magnetron sputtering system.

In the experiments, the chamber was first pumped down from 10^-6^ to 10^-7^ Torr and the voltage for sputtering was set at 28 V. The target was first pre-sputtered for 6 to 8 min for surface cleanness at 200 °C. Buffer layers were then created on the Si(100) substrates at 200 °C for 2 min at a deposition power of 150 W. The temperature of the heater was then raised to 400 °C. The target was pre-sputtered again for 3 min at 400 °C before a further 60 min of sputtering at 400 °C and at 150 W; thin films of Ti-doped ZnO at a certain thickness were formed. The films were deposited in high vacuum at different ambient atmospheres at a pressure of 1.0~2.0 mTorr: (1) a mixture of 85% Ar and 15% N_2_, (2) a mixture of 80% Ar and 20% O_2_, and (3) 100% Ar. The gas flow was set at 18.7~21.0 sccm (atm cm^3^/min). The target to substrate distance was fixed throughout the experiment. Two sets of samples were prepared at the 85% Ar and 15% N_2_ mixture ambient. The concentration of Ti doped in the doped samples was varied by changing the Ti plate size.

The thickness of Ti-doped ZnO films was measured using an Alpha-step 500 profilometer, revealing a thickness of approximately 500 nm. Scanning electron microscopy (SEM) images show a polycrystalline nature of the film surfaces, which have relatively small and slightly elongated spherical grain structures. X-ray fluorescence (XRF) analysis was also performed using white beam radiation at the Phase Contrast Imaging (PCI) beamline [20] at the Singapore Synchrotron Light Source (SSLS) to determine the composition of samples, as listed in Table 1. Zn_0.996_Ti_0.004_O and Zn_0.972_Ti_0.028_O were fabricated under 85% Ar and 15% N_2_, and Zn_0.933_Ti_0.067_O and Zn_0.994_Ti_0.006_O were fabricated under pure Ar, and 80% Ar and 20% O_2_, respectively.

**Table 1:** Composition, saturation magnetic moments M_s_ of Ti-doped ZnO films deposited on Si(100) substrate and the ambient atmosphere under which the films were fabricated.

Sample	Atmosphere	M_s_/*µ_B_* per Ti atom
Zn_0.996_Ti_0.004_O	Ar + N_2_	0.090 ± 0.004
Zn_0.994_Ti_0.006_O	O_2_ + N_2_	0.827 ± 0.013
Zn_0.933_Ti_0.067_O	Ar	0.036 ± 0.001
Zn_0.972_Ti_0.028_O	Ar + N_2_	0.039 ± 0.002

The XAFS spectra of the Ti-doped ZnO films were measured at the X-ray Development and Demonstration (XDD) beamline [21] at SSLS. The XDD beamline provides a photon energy range of 2.4–10 keV from a superconducting bending magnet. The XAFS spectra of reference samples were taken in transmission mode at room temperature. The film samples were measured in fluorescence mode using a Lytle-type detector with argon ambient flowing. Because of the low concentration and lower binding energy of Ti with respective to Zn, Ti K-edge fluorescence XAFS experiments were also repeated at the BL01B1 beamline [22,23] of SPring-8 using a 19-element solid state detector [24]. BL01B1 is a bending magnet beamline equipped with two mirrors and a fixed-exit double-crystal monochromator using Si(111) and Si(311) crystals. The energy was calibrated to the *K*-edge absorption of the Ti metal-foil. XAFS spectra were collected in the photon energy range from roughly 100 eV prior to the absorption edge to 800 eV above. The data presented here were collected at Spring-8.

Data analysis of XAFS follows a standard procedure. Because of the data statistics, only the two high Ti concentration samples were subject to the Fourier transform (FT). In the FT, the XAFS functions in a range of 2.9–11.0 Å^-1^ in *k* space were extracted, k^3^ weighted, and a Bessel window function was employed. A fit to the first shell was performed in the real space and the coordination number (CN), interatomic distance (R), and Debye-Waller (σ^2^) factor were extracted. *E*_0_ values were set as free variables during the fit. The inelastic factor, *s*_0_^2^, was fixed at 0.90.

A model 707 vibrating sample magnetometer produced by the Lake-Shore Company was used in the magnetic measurements of the film samples. The magnetic field was applied parallel to the plane of the film. Before the measurements, the sample holder with pure Si substrate was measured to calibrate the magnetic background. Magnetic contaminations by sample handling were avoided. A maximum of 5000 Gaus magnetic field was applied. All magnetization curves were measured at room temperature.

## 3. Results and Discussion

**Figure 1:**
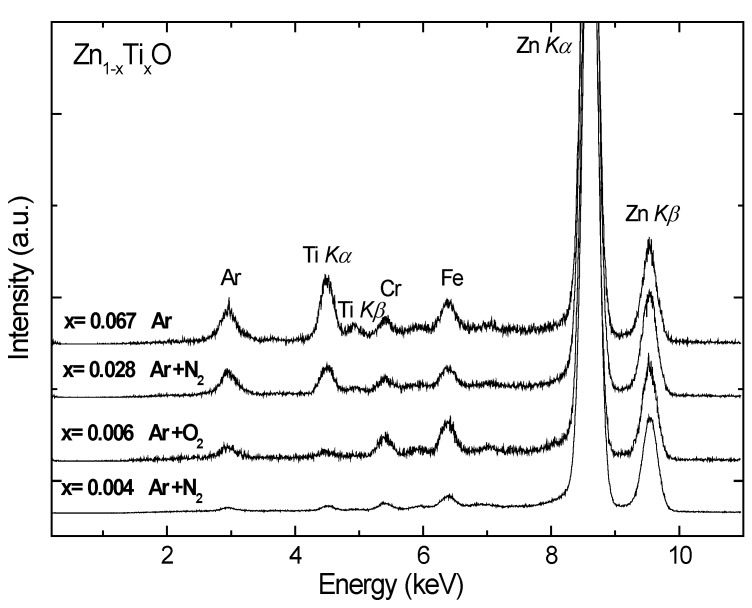
Normalized X-ray fluorescence spectra of the Zn_1-*x*_Ti*_x_*O films deposited on Si(100). The Ti compositions x relative to Zn were determined. The Fe and Cr peaks in the spectra are contaminants from the background that contain stainless steel and can be removed by careful shielding of radiation.

The penetration depth of the hard X-rays used in XRF is in the μm range; hence, XRF probes the bulk of the films. Figure 1 shows the normalized XRF spectra of the Ti-doped ZnO samples. The fluorescent peaks of Ti and Zn are clearly observable. The peak of argon at about 3 keV is from the rare gas since the samples were measured in air. The Cr and Fe signals are from the background contamination which contains stainless steel. By comparing the integrated areas under the Zn K_α_ and Ti K_α_ peaks and comparing with a calibrated sample of known concentration, the composition x of Ti was derived, as listed in Table 1.

**Figure 2:**
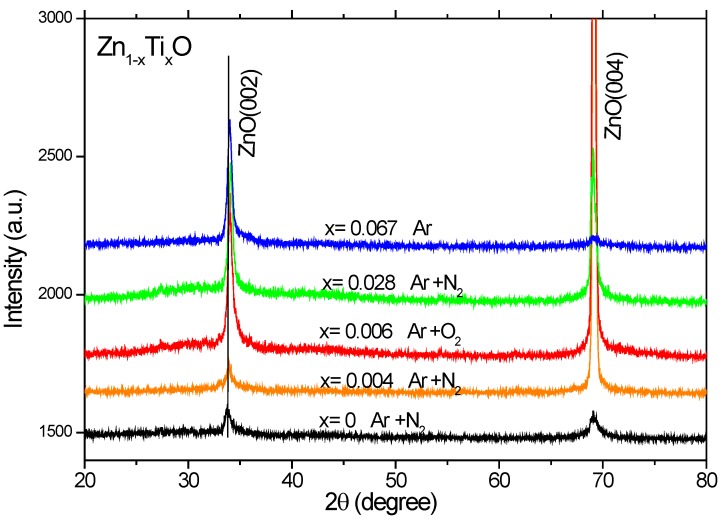
X-ray diffraction patterns of the Zn_1-*x*_Ti*_x_*O films deposited on Si(100).

X-ray diffraction (XRD) patterns were measured for the Ti-doped ZnO films (Figure 2), which show only (002) and (004) index peaks of ZnO, indicating that ZnO grains are preferentially c-axis oriented on Si(100). A strong crystallographic anisotropy is observed in the measured XRD patterns. No metal or metal oxide related peaks can be detected within the sensitivity of XRD. The peak position of ZnO:Ti is shifted by 0.1^o^–0.2^o^ towards higher angles as compared to the undoped ZnO, which implies a decreased lattice parameter c for the Ti-doped ZnO films. The decrease in c is in agreement with the previous theoretical calculations [14]. This might be due to the atomic radius difference for Ti and Fe ions and complex defect formation [25,26].

**Figure 3:**
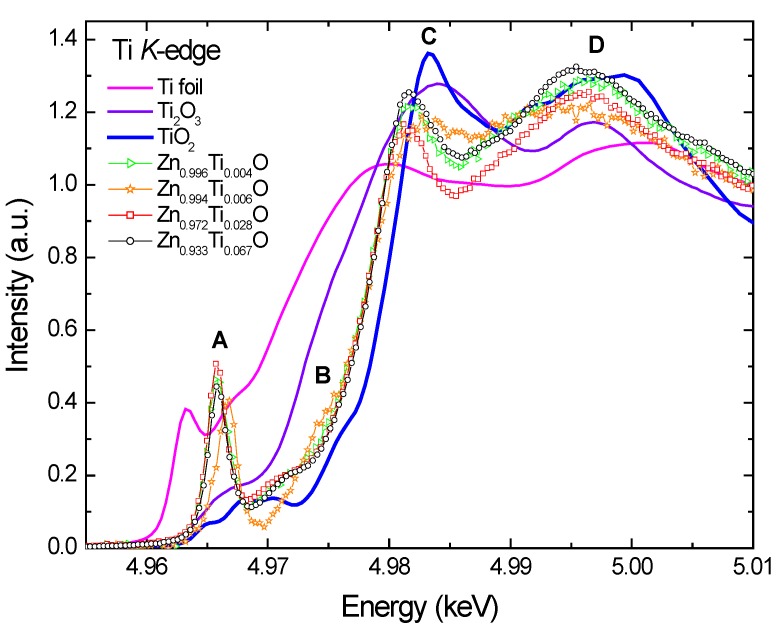
Normalized Ti *K*-edge XANES of Ti-doped ZnO films and reference samples, Ti foil, Ti_2_O_3_ and TiO_2_.

Figure 3 compares the normalized Ti *K*-edge X-ray absorption near-edge spectroscopy (XANES) spectra of the Ti-doped ZnO films with those of Ti foil, Ti_2_O_3_, and TiO_2_. Several main features are identified and marked: a very sharp pre-edge peak A, a shoulder peak B, a main peak C, and a broad peak D at the post-edge region. Generally, the peak A for transitional 3*d* metal oxides is interpreted as a quadrupolar electronic transition from 1*s* to the unoccupied 3*d* final states hybridized with the 4*p* character of the absorber; its intensity is enhanced by the local atomic configuration that lacks centrosymmetry [27,28].

The XANES spectra of Ti doped ZnO samples display significantly different structures from the reference Ti foil and oxides. The very prominent pre-edge peak indicates a tetrahedral Ti-O coordination, implying a substitutional incorporation of Ti ions into the tetrahedral zinc sites in the wurzite ZnO lattice. The spectra of Ti_2_O_3_ and TiO_2_ show only a weak pre-edge peak A due to the octahedral local structures around Ti ions [29,30,31]. The pre-edge features are insensitive to Ti-O bond length, but are sensitive to valence, occurring about 2.0 eV lower in Ti^3+^ samples compared to Ti^4+^ [32]. However, it is difficult to deduce unambiguously the Ti valence because the pre-edge features vary in both position (~2 eV) and normalized height as a function of Ti coordination (4, 5 or 6 oxygen nearest neighbors) [33]. Ideally the Ti doped samples should be compared to the references with identical coordination and known valency. Few minerals of Ti in nature appear to have major Ti^3+^ [32].

There is an evident shift of the peak A to higher energy for Zn_0.994_Ti_0.006_O relative to the rest of the samples. Since the feature A is still predominant, it implies that the majority of Ti in Zn_0.994_Ti_0.006_O is still tetrahedrally coordinated. This chemical shift is either due to a change in the valence state of Ti to a higher oxidation state or due to the precipitation of a portion of octahedral Ti species. The slightly reduced peak height for this sample implies the latter case is more likely. Some other differences are also observed: the shoulder B is more pronounced, and the peak C broadened, with its intensity much lower. Although the Ti doping concentration is extremely low in this sample, a portion of titanium oxides or zinc titanate might have formed during the sputtering process. The Ti atoms sputtered from the target might have reacted actively with oxygen ions and formed oxide precipitates before the deposition. This would hinder them from incorporating into the ZnO lattice. Despite the similar deposition rate for the x = 0.004 and x = 0.006 samples, they are not prepared in the same ambient atmospheres. This leads to the different the species for Ti, indicating that the prepared Ti speciation in ZnO is sensitive to the ambient atmosphere. These trace precipitates containing octahedral Ti may be too dispersive and disordered, and are not detectable by XRD.

**Figure 4:**
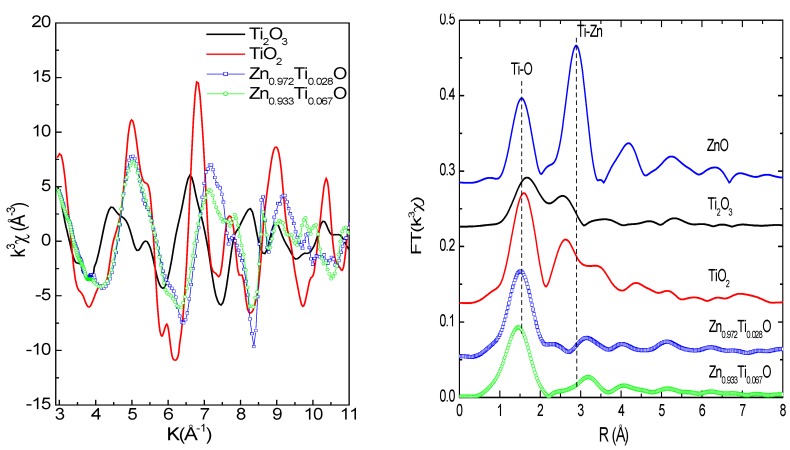
(a; left): Ti *K*-edge EXAFS functions; (b; right): Fourier Transform magnitudes (vertically shifted) of the Ti-doped ZnO films and reference Ti oxide samples. Phase shift was not corrected.

Figure 4a and Figure 4b show Ti *K*-edge EXAFS functions and Fourier transform of the Zn_0.972_Ti_0.028_O and Zn_0.933_Ti_0.067_O films and reference samples. The Ti *K*-edge EXAFS functions for the low concentration samples (Zn_0.996_Ti_0.004_O and Zn_0.994_Ti_0.006_) are not usuable because of the poor statistics. The EXAFS functions for Zn_0.972_Ti_0.028_O and Zn_0.933_Ti_0.067_O are very similar and different from Ti oxides. In Figure 4b, the first peak at 1.4~1.5 Å (phase shift was not corrected) is due to the Ti-O (or Zn-O for ZnO) coordinations in the first shell. The second peak at about 3.15~3.2 Å corresponds to the second shell, which contains 12 Zn and 1 O atoms for ZnO [34]. The second coordination peaks are weak for the Ti-doped samples, but not absent if one looks into the structures at an even higher R, which represents the level of noise. Even at such low Ti concentrations, an extreme disorder is possible because of the large difference in atomic radius of Zn^2+^ (0.600 Å) and Ti^4+^ (0.42 Å) in a tetrahedral Ti-O coordination [35]. A shift towards higher *R* for the second Ti-Zn shell is also observed for the Ti doped samples relative to ZnO.

**Figure 5:**
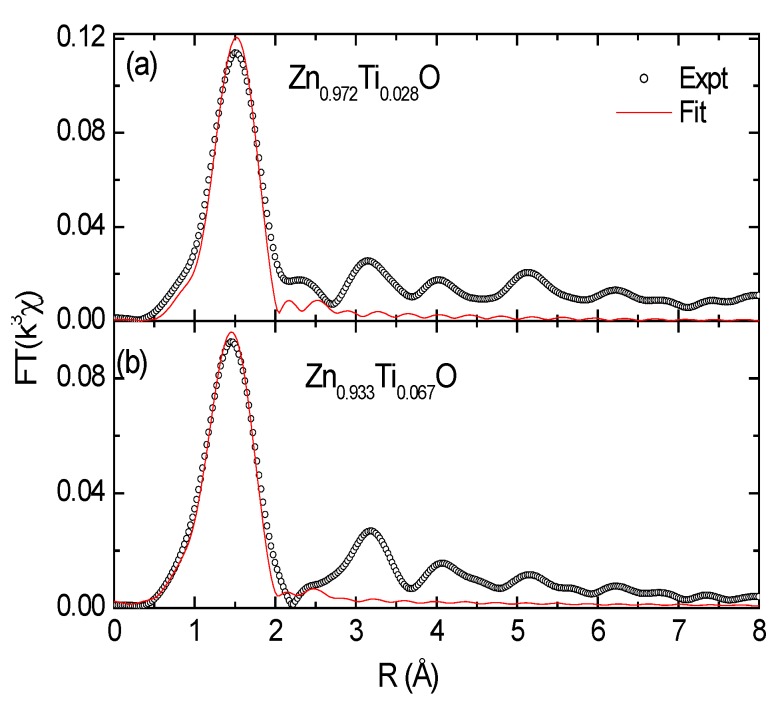
Theoretical fit to the first peak in the real space for (a) Zn_0.972_Ti_0.028_ and (b) Zn_0.933_Ti_0.067_O films.

Data fit of the Ti-doped samples was performed in real space for the first shell (Figure 5); the results are tabulated in Table 2. The radial structural parameters for the reference samples are extracted from crystallographic data. The fit yields a Ti-O distance of 1.87–1.89 Å for the Ti doped ZnO samples. It is much less than those of Zn-O (1.97 Å) in ZnO and Ti-O in Ti_2_O_3_ and TiO_2_, as listed in Table 2. This much shorter Ti-O bond favors the 4+ valence and a tetrahedral coordination, since the difference in bond length for Ti^3+^ and Ti^4+^ is 0.05–0.1 Å [32] and the atomic radius in tetrahedral sites is shorter than in octahedral sites [35]. The short Ti-O distance implies strong local interaction in Ti-O in the ZnO matrix. The empty *d*-orbitals of Ti might be involved in the chemical bonding. Similarly, a strong Co-O local interaction in Co-doped ZnO nanoparticles has been observed [36]. It is in agreement with our XRD results and the theoretical calculations performed by Xiong *et al.* [12]. The coordination number (CN) for Zn_0.972_Ti_0.028_O is close to 4 and it gets higher (5.4) for the x = 0.067 doped sample, implying a portion of precipitates with higher CN at the first shell may exist in the 6.7% doped concentration. Two other possible zinc titanates that may form precipitates are Zn_2_Ti_3_O_8_ and ZnTiO_3_. The radial structures for the first shell are extracted from the crystallographic data and are also listed in Table 2. Zn_2_Ti_3_O_8_ is a metastable compound and possesses a defect spinel structure [37]. Ti^4+^ ions in Zn_2_Ti_3_O_8_ occupy octahedral sites, whereas part of the Zn^2+^ ions occupy octahedral sites and the rest are in tetrahedral sites. ZnTiO_3_ has a perovskite structure and is stabilized by the six-fold coordination of the Ti^4+^ ions and 12-fold coordination of the Zn^2+^ ions [38].

The shorter Ti-O and larger Ti-Zn(Ti) distances at the first and second shells imply an amorphous-like local structure [39], *i.e.*, a highly ordered local structure. The presence of some TiO_4_ clusters observed in Cu doped ZnO cannot be ruled out [40]. In fact, the boundary for substitutional doping in ZnO and clustering becomes ambiguous if the intermediate range ordering is missing. The substitution of Ti in the Zn sites has induced a large distortion in the ZnO lattice.

**Table 2:** Structural parameters of references from the literatures and results of the data fit of the Ti-O or Zn-O(ZnO) shell in Zn_0.972_Ti_0.028_O and Zn_0.933_Ti_0.067_O films. CN = coordination number; *R* (Å) = bond length; and σ^2^ (Å^2^) = Debye-Waller factor. The uncertainties are 10%, 0.02 Å, and 10%, respectively.

Sample	CN	R	σ^2^
ZnO	4	1.97	--
Ti_2_O_3_	6	2.05	--
Anatase TiO_2_	6	1.95	--
Rutile TiO_2_	6	1.96	--
Zn_0.972_Ti_0.028_O	4.3	1.89	0.0044
Zn_0.933_Ti_0.067_O	5.4	1.87	0.0087
Zn_2_Ti_3_O_8_	6	2.10	
ZnTiO_3_	6	2.01~2.06	--

**Figure 6:**
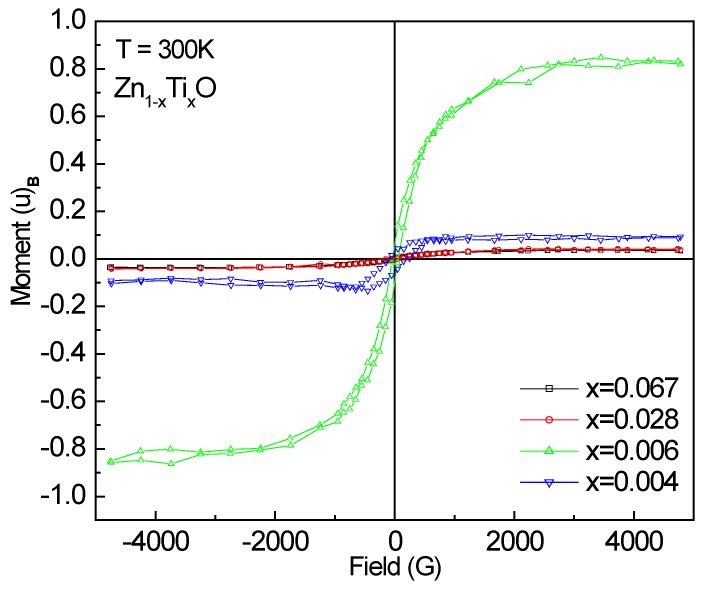
M-H loops measured at room temperature for Ti-doped ZnO films.

The M-H magnetization (*µ_B_*/Ti) curves measured at room temperature are shown in Figure 6 for the doped samples. All films are ferromagnetic as observed from the M-H hysteresis loops. The saturation magnetization Ms (*µ_B_*/Ti) values were derived and listed in Table 1. The largest magnetic moment is 0.827 ± 0.013 *µ_B_*/Ti for Zn_0.994_Ti_0.006_O, several times larger than that (0.15 *µ_B_*/Ti) reported by Antony *et al.* [13] and Venkatesan *et al.* [8] at room temperature for 5% Ti-doped ZnO sample. This is astonishing. One can note that the two 0.6% and 0.4% samples with similar doping concentrations show a large difference in the magnetic moment. The XANES spectra have shown a majority of tetrahedral Ti-O coodination and a portion of Ti species with higher Ti-O coordinations in the 0.6% doped sample. However, the unusually high ferromagnetism might not be attributed to the precipitates, since none of them are ferromagnetic.

The Ti-doped ZnO thin film samples in the present study are insulators; hence, the hole-mediated mechanism is unlikely to interpret the observed ferromagnetism. Recent studies have shown that intrinsic defects in ZnO play an important role in the room-temperature ferromagnetism in transition metal doped ZnO, in particular, with the cationic vacancies [41,42,43]. Similar explanations may also be applicable for the Ti-doped ZnO samples. High magnetic moment for the 80% Ar, 20% O_2_ ambient sample indicates the ferromagnetism may not be oxygen vacancy relevant because in an oxygen-rich atmosphere, it is not favorable for oxygen vacancy formation. The ferromagnetic ordering may be improved by the cationic vacancy-derived impurity band. Similar to Li doped ZnO, the formation energy of Zn vacancies can be lowered by the doping of Ti by forming defect complexes [43]. A higher Ti valence state than Zn^2+^ implies that Ti atoms may act as donors by supplying electrons or they may favor the creation of more Zn vacancies (V0 centers) [12]. It is clear that more experiments and theoretical calculations are necessary to provide direct evidence.

## 4. Conclusions

Zn_1-*x*_Ti*_x_*O (x = 0.004, 0.006, 0.028, 0.067) films were fabricated on Si substrates by radio-frequency magnetron sputtering. Ferromagnetism was measured at room temperature. A strong ambient condition dependence of the structures and magnetism was observed; the film sample prepared at 80% Ar, 20% O_2_ ambient shows the largest magnetic moment (0.827 ± 0.013 *µ_B_*/Ti). The local environment of Ti was investigated by measuring XAFS at Ti K-edge. The results indicate that the majority of Ti ions take the tetrahedral crystallographic sites and are incorporated into the substitutional sites in the ZnO lattice. At higher concentration doping (6.7%), some precipitates of Ti-O speciation may exist. The FT spectra and fit results indicate a strong Ti-O interaction and the Ti doping induces a large distortion because of the lattice mismatch. The preparation at different ambient conditions may change charge equilibrium in the Ti-doped ZnO and hence affect the Zn vacancy concentration.
